# Association of Visceral Fat Area and Hyperuricemia in Non-Obese US Adults: A Cross-Sectional Study

**DOI:** 10.3390/nu14193992

**Published:** 2022-09-26

**Authors:** Zhiying Li, Lijie Gao, Xiaoqing Zhong, Guanrui Feng, Fengqiu Huang, Sujian Xia

**Affiliations:** Department of Public Health and Preventive Medicine, School of Medicine, Jinan University, Guangzhou 510632, China

**Keywords:** hyperuricemia, visceral fat area, uric acid, NHANES

## Abstract

This study aimed to investigate the relationship between visceral fat area (VFA) and hyperuricemia (HUA) among non-obese adults. We extracted data from 6224 US adults aged 20–59 years from the National Health and Nutrition Examination Survey (NHANES) from 2011–2018. The VFA was divided into four quartiles (Q1–Q4). We used multivariable logistic regression models to control for known confounders. A generalized additive model (GAM) and restricted cubic spines were used to examine the association between VFA and HUA stratified by sex, and a two-piecewise linear regression model was used to calculate the threshold effect among males. The results revealed that the prevalence of HUA was 11.8% (men 15.8%, women 7.2%). In the fully adjusted model, there was a positive association between VFA and HUA [as a quartile variable, Q4 vs. Q1, odds ratio (OR): 3.77 and 95% confidence interval (CI): (2.47~5.75), *p* < 0.001, *p* for trend < 0.001; as a continuous variable, per 10 cm^2^ increment, OR (95%CI):1.10(1.07,1.14), *p* < 0.001]. Besides, this positive association remained significantly stratified by sex. Interestingly, we observed a nonlinear dose-response relationship between VFA and HUA in males (inflection point: 107.46 cm^2^). In conclusion, our study confirmed a significant positive relationship between VFA and HUA among non-obese adults and remained statistically significant when stratified by sex.

## 1. Introduction

In recent years, HUA has become a common metabolic disease worldwide [1], characterized by elevated levels of uric acid (UA) in the blood, which affects patients of all ages and genders [2]. Studies have shown that the global incidence of HUA has been on the rise since the 1960s, and up to 2016, the global incidence of HUA has been as high as 21% [1]. The incidence rate in the United States ranges from 14.6% to 20% [3]. A large number of studies have shown that HUA is associated with hypertension, insulin resistance, liver dysfunction, dyslipidemia, ventilation arthritis, chronic kidney disease (CKD), cardiovascular diseases, and other diseases [4,5,6]. The increasing incidence of these diseases has greatly increased the public health burden of society as a whole [7].

At present, research on the factors related to HUA has been on the rise [8,9]. There is no doubt about the relationship between obesity and HUA, and obesity is a risk factor for HUA [10]. In addition, many studies have shown that visceral fat has a close relationship with HUA [11,12]. People have gradually realized that an elevated visceral fat content in non-obese people can also lead to a series of diseases [13,14]. One study suggests that increased visceral fat may increase the risk of atherosclerosis in non-obese Japanese individuals [15].

HUA is not uncommon in non-obese people, and the excess of visceral adipose tissue in non-obese people may also affect the occurrence of HUA [16]. However, little information is available on the association between HUA and visceral fat in non-obese individuals. Therefore, this study aimed to explore the relationship between VFA and HUA in non-obese people.

## 2. Materials and Methods

### 2.1. Study Population

**Figure 1:**
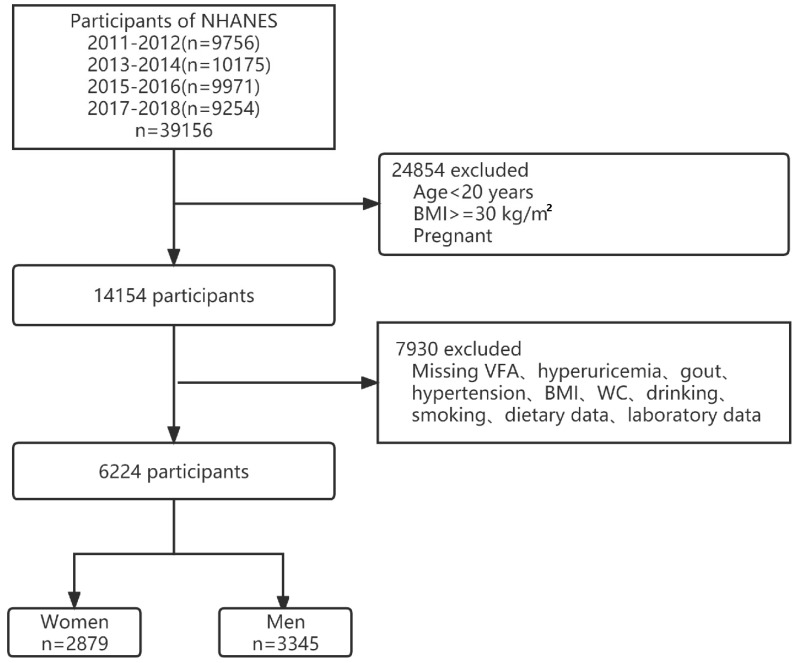
Flowchart of participant selection. Abbreviations: VFA, visceral fat area; BMI, body mass index; WC, waist circumference.

The NHANES is a nationally representative cross-sectional survey designed to measure the health and nutritional status of adults and children in the United States and is administered by the Centers for Disease Control and Prevention (CDC). NHANES is a dataset accessible to the public that resides in the public domain (http://www.cdc.gov/nchs/nhanes/ (accessed on 10 July 2022)). All subjects provided written informed consent, and all procedures were approved by the NCHS Research Ethics Review Board. For our study, we utilized the four NHANES cycles from 2011 to 2018. The participants were invited for examination visits where blood and urine samples were collected following a home interview. The 2011–2018 NHANES included 39,156 participants, and our analyses were confined to 22,617 participants aged 20 years and above. We excluded people with a body mass index (BMI) of ≥30 kg/m^2^, pregnant women, and those with missing important variable data. Finally, our analysis included 6224 participants (3345 women and 2879 men) (Figure 1).

### 2.2. Exposure Variable and Outcomes

The exposure variable, VFA, was measured by a dual-energy X-ray absorptiometry (DXA) scan of the entire body using a QDR-4500 Hologic Scanner (Bedford, MA, USA). The serum uric acid (SUA) concentration was detected on a Beckman Coulter UniCel^®^ DxC800 from 2008–2016 and a Roche Cobas 6000(c501 module) in 2017 and 2018. The HUA was defined by the cut-off values of 7.0 mg/dL for men and 6.0 mg/dL for women [17].

### 2.3. Covariates

The sociodemographic covariates included age (years), sex, race/ethnicity (non-Hispanic white, non-Hispanic black, Mexican American, and others), marital status (married, living with a partner, or living alone), and education attainment (less than high school, completed high school, and more than high school). The health-related covariates included BMI, waist circumference (WC), smoking status, drinking status, and vigorous recreational activity (yes or no). Venous blood samples were taken to measure the serum levels of total cholesterol (TC), hemoglobin A1c (HbA1c), high-density lipoprotein cholesterol (HDL-C), creatinine (Cr), triglycerides (TG), and albumin-to-creatinine ratio (ACR). The BMI was computed as the weight divided by the square of the height (kg/m^2^). Individuals who had never smoked 100 cigarettes in their lifetimes were classified as never smokers; subjects who had smoked 100 cigarettes in their lifetimes were classified as former smokers if they answered “No” to the question “Do you smoke now?” and as current smokers if they answered “Yes” [18]. The drinking status was categorized as never (<12 drinks in any one year), former (≥12 drinks in any one year and not drinking now), and current (≥12 drinks in any one year and drinking now).

The medical history included gout, diabetes, hypertension, and CKD. The presence of gout was determined based on the self-reported answer to the question “Has a doctor or other health professional ever told you that you have gout?”. Diabetes was defined as a fasting plasma glucose level of ≥7.0 mmol/L, a glycohemoglobin level of ≥6.5%, the use of diabetes medication or insulin, or a self-reported diagnosis of diabetes [19]. Hypertension was defined as a mean systolic blood pressure of ≥130 mmHg, a mean diastolic blood pressure of ≥80 mmHg, having a self-reported hypertension diagnosis, or using an antihypertensive drug [20]. The CKD was defined as an estimated glomerular filtration rate (eGFR) of <60 mL/min/1.73 m^2^ and/or albuminuria [21]. The eGFR was calculated using the Chronic Kidney Disease Epidemiology Collaboration Equation [22]. The dietary intakes of energy and nutrients (protein, carbohydrate, fiber, and fat) were gathered via two 24-h dietary recall interviews. If an individual completed both of the 24-h recalls, the average dietary intake from the two recalls was used. Otherwise, we took the data from a single dietary recall [23]. There are descriptions of all the variables and acquisition processes on the website (www.cdc.gov/nchs/nhanes/ (accessed on 10 July 2022).

### 2.4. Statistical Methods

The data were divided into the following two categories: continuous variables and categorical variables. Based on the normality of their distribution, continuous variables were further classified into two categories. The normally distributed continuous variables were presented as a mean ± standard deviation (SD), while the non-normally distributed variables were presented as a median ± interquartile range (IQR). The statistical differences in each group were calculated by a Student’s *t*-test or a Mann–Whitney U test (continuous variables). The categorical variables were presented as percentages and compared using the chi-square test. Multivariable logistic regression analyses were used to investigate the association between VFA and HUA, and the lowest quartile was used as the reference category. Then, stratified analyses were conducted by sex to determine the associations between VFA and SUA, and the results are presented as an OR with 95% CI. Based on previous research and a changed effect of interest by >10% in this study population [24], three models were used as follows: model 1, adjusted for age and race; model 2, adjusted for age, race, marital status, BMI, WC, smoking status, drinking status, vigorous recreational activity, and carbohydrate intake; model 3, adjusted for age, race, marital status, BMI, WC, smoking status, drinking status, vigorous recreational activity, carbohydrate intake, TG, ACR, HbA1c, HDL-C, gout, and hypertension.

Additionally, we used a GAM and restricted cubic splines to explore the nonlinear relationship between VFA and HUA [25] stratified by sex. If a nonlinear association was observed, we constructed a two-piecewise linear regression model to calculate the threshold effect. When the VFA and HUA become apparent in the smoothed curve, the recursive technique automatically calculates the threshold, where the maximum model likelihood will be used [26]. The software packages R (http://www.R-project.org (accessed on 15 May 2022), The R Foundation) and Free Statistics software version 1.6 were used to perform all of the statistical analyses. The statistical differences were considered significant at *p* < 0.05 (two-sided).

## 3. Results

### 3.1. Baseline Characteristics of the Participants

The baseline characteristics of the participants were presented by VFA quartiles as follows: Q1 ≤ 48.24 cm^2^; Q2: 48.24~71.64 cm^2^; Q3: 71.64~104.06 cm^2^; Q4: ≥104.06 cm^2^. The mean participant age was 38.6 ± 11.8 years, and 3345 (53.7%) of the participants were men. The median (IQR) VFA was 71.6 cm^2^ (48.2 cm^2^, 104.1 cm^2^). The VFA level was positively related to the following variables: age, race/ethnicity, marital status, education attainment, BMI, WC, TC, SUA, HbA1c, HDL-C, Cr, TG, ACR, smoking status, drinking status, vigorous recreational activity, and dietary intake of energy and nutrients (protein, fiber, and fat). Moreover, subjects with a higher VFA were more prone to having diabetes, hypertension, gout, and CKD. Interestingly, higher VFA levels were associated with lower levels of eGFR (Table 1).

### 3.2. Incidence of HUA

The overall prevalence of HUA was 11.8%. In males, the mean ± SD SUA level was 5.8 ± 1.2 mg/dL, the median VFA level was 79.8 cm^2^ (IQR: 53.9 cm^2^, 112.6 cm^2^), and the prevalence of HUA was 15.8%. In females, the mean ± SD SUA level was 4.4 ± 1.0 mg/dL, the median VFA level was 64.0 cm^2^ (IQR: 39.7 cm^2^, 94.0 cm^2^), and the incidence of HUA was 7.2% (Table 2).

### 3.3. VFA and HUA

The associations between VFA and HUA by multivariate logistic regression analyses and subgroup analyses stratified by sex are shown in Table 3. After an adjustment for different confounders, the positive relationships between the VFA and the risk of HUA were found as a quartile variable, Q4 vs. Q1, OR (95%CI): 3.77 (2.47~5.75), *p* < 0.001, *p* for trend < 0.001; as a continuous variable, per 10 cm^2^ increment, OR (95%CI): 1.10 (1.07,1.14), *p* < 0.001. Besides, among men, per 10 cm^2^ increment in VFA, the risk of SUA increased by 6% [OR (95%CI): 1.06 (1.02~1.11)] in the fully adjusted model. When the VFA was divided into four groups according to quartiles, we compared the Q1 with the adjusted OR of Q2, Q3, and Q4, which were 1.15 (95% CI:0.80~1.65), 1.88 (95% CI:1.25~2.84), and 2.04 (95% CI:1.24~3.38) in model 3. Among women, per 10 cm^2^ increment in VFA, the risk of SUA increased by 19% [OR (95%CI): 1.19 (1.12~1.27)] in the fully adjusted model. The multivariable-adjusted OR for HUA compared Q1 with Q2, Q3, and Q4, which were 2.12 (95% CI:1.13~3.97), 2.50 (95% CI:1.26~4.95), and 5.51 (95% CI:2.52~12.03). The highest level of VFA was associated with an increased risk of HUA.

### 3.4. The Nonlinear Relationship between VFA and HUA

**Figure 2:**
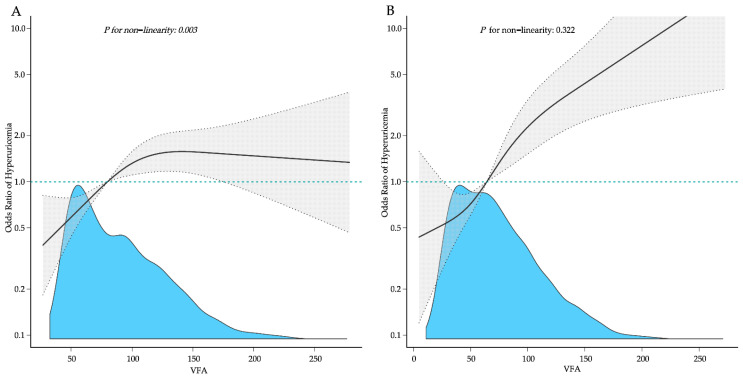
Adjusted dose-response association between the VFA and the risk of HUA with a restricted cubic spline function. (**A**) Males; (**B**) Females. Model with three knots located at the 5th, 50th, and 95th percentiles. The dashed lines represent 95% confidence intervals.

We observed a nonlinear dose-response relationship between the VFA and HUA in males (Figure 2A). By using a two-piecewise linear regression model, we found that the threshold of the VFA was 104.79 cm^2^ (Table 4). Moreover, in males with a VFA of <104.79 cm^2^, every 1 cm^2^ increase in the VFA increased the risk of SUA by 1.5% (95% CI: 1.005~1.025). By contrast, in males with a VFA of ≥104.79 cm^2^, a 1 cm^2^ increase in the VFA increased the risk of HUA by 0.2%, but the *p* value indicated no statistical significance (95% CI: 0.995~1.010). The dose-response relationship between the VFA and HUA in females (Figure 2B) was positive in a linear manner (*p* for nonlinearity = 0.322).

## 4. Discussion

Our research, which is based on analyzing the NHANES data from 2011–2018, indicated that VFA was significantly and positively associated with HUA in non-obese persons aged 20–59 years. In the fully adjusted models, the analysis stratified by sex revealed that the VFA remained positively and substantially linked with HUA. Additionally, the non-linear relationship between the VFA and HUA was identified in males with the inflection point at 104.79 cm^2^.

Several epidemiological studies have suggested that obesity plays an important role in HUA [10,27]; however, the function of body fat distribution in UA metabolism remains unclear. Consistent with previous research [28,29], we demonstrated that visceral fat is strongly related to many health conditions, including HUA, diabetes, hypertension, gout, and CKD. According to a study by Huang et al. [11], the visceral adipose increase was strongly linked to HUA in Chinese people, but the research is only limited to middle-aged and elderly people. Likewise, a cross-sectional study of 862 individuals undergoing medical examinations revealed that visceral fat or liver fat were substantially related to HUA [12]; yet, the study population consisting solely of men was the most significant restriction of the research design. Traditional anthropometric markers, such as BMI, WC, and WHR, have their drawbacks in assessing visceral obesity, which is vulnerable to race, gender, and age [30,31]. In our study, a DXA was utilized to evaluate the android to gynoid fat ratio and visceral adipose tissue.

With the development of material living conditions and the rise of unhealthy lifestyles, the incidence rate of HUA has maintained its generally upward trend. The data from NHANES implied an almost 20% HUA prevalence among the US general population during 2015–2016 [3]. In our study, the prevalence of HUA was 11.8% among non-obese individuals. Therefore, non-obese individuals with HUA should also be of concern. The reason why non-obese individuals still have HUA risk may be related to visceral obesity. Until now, only one study [16] demonstrated the association of metabolic score for visceral fat and HUA in non-obese adults, but this index does not accurately reflect the VFA. Therefore, there were still limited studies on the relationship between VFA and HUA in non-obese people. In our study, we found that VFA in individuals without obesity was positively associated with the risk of HUA.

The underlying mechanism may be the excess free fatty acids produced from visceral fat, which are causing a metabolic imbalance induced by UA on the kidneys and the liver [32]. Extra visceral fat alters the mitochondrial function and energy metabolism of tumor cells [33] and changes the expression of genes connected with inflammation in the peripheral blood cells [34]. Visceral adipose tissues may present varying metabolic risks [35]. They are metabolically active and modulate many adipocytokines linked to insulin resistance [36]. Insulin resistance or HUA can enhance UA reabsorption from the renal tubules, hence, decreasing urine UA excretion and increasing plasma UA concentrations [37]. In addition, the increased release of UA from the adipose tissues suggests the existence of a vicious circle between UA and fat mass gain [38]. Moreover, our study also observed that VFA was negatively associated with eGFR [39], and a lower eGFR may cause UA underexcretion [40]. Therefore, the mechanism through which VFA induces HUA remains to be revealed.

Interestingly, according to a prospective study [41], we also observed that UA levels were significantly higher in males than females, most likely due to the effect of estrogen on enhancing the renal clearance of UA [42]. Specifically, it was discovered that estrogen decreases the urate reabsorptive transporter expression at the posttranscriptional level, resulting in higher UA excretion and decreased SUA levels [43]. Thus, estrogen may explain the various patterns of relationships between the VFA and HUA in different sexes. Other studies suggest that females may be more susceptible to UA-induced organ damage [44,45], which may explain why UA levels in females were within a lower range than in males. However, further research is required to determine the precise mechanisms. In our research, the non-linear associations between VFA and HUA varied between sexes. The prevalence of HUA increased steadily with the increase in VFA, but an inflection point was identified in males. Therefore, it may be necessary to consider sex differences to prevent HUA in individuals without general obesity.

This study has several advantages. First, this is the first study to assess the relationship between the VFA and the risk of HUA among non-obese US adults. In addition, we selected a relatively large sample size. On the other hand, our study has some limitations. First, we used the primary definition of HUA in this study. However, recent studies indicate that the definition of HUA, particularly asymptomatic HUA in the presence of CKD, is indeed controversial [46,47]. Second, although the data for the VFA and HUA levels in our study are objective, some covariates and medical history were identified as self-reported; therefore, the results of this study may be affected by bias. Third, considering ethnic differences, it is uncertain whether the findings of this study can be applied to other ethnic groups. Fourth, due to the nature of the cross-section data, this study only proves the correlation between the VFA and HUA but cannot prove a cause–effect relationship. Finally, we acknowledge that the gender differences reported in this paper may be influenced by women’s menstrual status and other outcome factors not explicitly accounted for in our model. Further studies should focus on identifying the causal relationship between VFA and HUA.

In conclusion, our findings confirmed a strong positive correlation between VFA and HUA in non-obese adults, even after correcting for a large number of potential confounding variables. Additionally, when stratified by sex, this connection remained statistically significant. However, further longitudinal investigations are required to corroborate our findings in external populations and evaluate the underlying mechanisms.

## Figures and Tables

**Table 1:** Baseline characteristics of the study participants according to VFA.

Variables	Total (*n* = 6224)	Q1 (*n* = 1556)	Q2 (*n* = 1556)	Q3 (*n* = 1556)	Q4 (*n* = 1556)	*p* Value
Sex, n (%)						<0.001
Male	3345 (53.7)	578 (37.1)	870 (55.9)	881 (56.6)	1016 (65.3)	
Female	2879 (46.3)	978 (62.9)	686 (44.1)	675 (43.4)	540 (34.7)	
Age, years	38.6 ± 11.8	31.4 ± 10.3	35.5 ± 10.9	40.3 ± 10.6	47.2 ± 8.8	<0.001
Race/ethnicity, %						<0.001
Non-Hispanic White	2316 (37.2)	573 (36.8)	599 (38.5)	539 (34.6)	605 (38.9)	
Non-Hispanic Black	1131 (18.2)	424 (27.2)	326 (21.0)	242 (15.6)	139 (8.9)	
Mexican-American	1388 (22.3)	226 (14.5)	298 (19.2)	382 (24.6)	482 (31.0)	
Other	1389 (22.3)	333 (21.4)	333 (21.4)	393 (25.3)	330 (21.2)	
Marital status, %						<0.001
Married or living with partner	3665 (58.9)	693 (44.5)	854 (54.9)	1018 (65.4)	1100 (70.7)	
Living alone	2559 (41.1)	863 (55.5)	702 (45.1)	538 (34.6)	456 (29.3)	
Education attainment, %						<0.001
Less than High school	1044 (16.8)	181 (11.6)	261 (16.8)	252 (16.2)	350 (22.5)	
Completed High school	1287 (20.7)	295 (19.0)	305 (19.6)	339 (21.8)	348 (22.4)	
More than High school	3893 (62.5)	1080 (69.4)	990 (63.6)	965 (62.0)	858 (55.1)	
VFA, cm^2^	71.6 (48.2, 104.1)	37.5 (29.3, 43.0)	59.1 (53.5, 65.1)	87.1 (79.2, 94.8)	128.7 (115.5, 147.5)	<0.001
BMI, kg/m^2^	24.8 ± 3.2	21.9 ± 2.8	24.2 ± 2.7	25.9 ± 2.4	27.2 ± 2.0	<0.001
WC, cm	88.2 ± 9.7	77.8 ± 6.6	85.8 ± 6.4	91.6 ± 6.4	97.6 ± 6.2	<0.001
Smoking status, %						<0.001
Never	3754 (60.3)	1024 (65.8)	959 (61.6)	955 (61.4)	816 (52.4)	
Current	1028 (16.5)	181 (11.6)	221 (14.2)	275 (17.7)	351 (22.6)	
Former	1442 (23.2)	351 (22.6)	376 (24.2)	326 (21.0)	389 (25.0)	
Drinking status, %						0.696
Never	745 (12.0)	176 (11.3)	185 (11.9)	196 (12.6)	188 (12.1)	
Current	677 (10.9)	155 (10.0)	168 (10.8)	177 (11.4)	177 (11.4)	
Former	4802 (77.2)	1225 (78.7)	1203 (77.3)	1183 (76.0)	1191 (76.5)	
Vigorous recreational activity, %						<0.001
Yes	2269 (36.5)	748 (48.1)	659 (42.4)	520 (33.4)	342 (22.0)	
No	3955 (63.5)	808 (51.9)	897 (57.6)	1036 (66.6)	1214 (78.0)	
TG, mg/dL	103.0 (68.8, 164.0)	73.0 (53.0, 99.0)	90.0 (63.0, 132.0)	119.0 (79.0, 180.0)	160.0 (107.0, 243.0)	<0.001
HbA1c, %	5.5 ± 0.8	5.2 ± 0.5	5.4 ± 0.8	5.5 ± 0.8	5.8 ± 1.1	<0.001
TC, mg/dL	190.0 ± 40.2	174.3 ± 33.5	183.5 ± 36.5	196.0 ± 40.8	206.2 ± 41.8	<0.001
SUA, mg/dL	5.1 ± 1.3	4.6 ± 1.1	5.1 ± 1.2	5.3 ± 1.4	5.6 ± 1.4	<0.001
HDL-C, mg/dL	55.3 ± 16.2	62.5 ± 15.6	57.6 ± 16.3	52.9 ± 15.1	48.2 ± 14.3	<0.001
Cr, mg/dL	0.8 (0.7, 1.0)	0.8 (0.7, 0.9)	0.8 (0.7, 1.0)	0.8 (0.7, 1.0)	0.8 (0.7, 1.0)	<0.001
eGFR, ml/minute/1.73 m^2^	103.7 ± 18.2	109.6 ± 18.3	106.1 ± 17.9	102.3 ± 17.2	96.8 ± 17.1	<0.001
ACR, mg/g	6.1 (4.2, 10.0)	6.3 (4.3, 10.3)	5.7 (4.0, 9.1)	5.8 (4.1, 9.4)	6.5 (4.4, 11.1)	<0.001
Gout, %						<0.001
No	6124 (98.4)	1550 (99.6)	1542 (99.1)	1525 (98.0)	1507 (96.9)	
Yes	100 (1.6)	6 (0.4)	14 (0.9)	31 (2.0)	49 (3.1)	
Diabetes, %						<0.001
No	5876 (94.4)	1544 (99.2)	1513 (97.2)	1479 (95.1)	1340 (86.1)	
Yes	348 (5.6)	12 (0.8)	43 (2.8)	77 (4.9)	216 (13.9)	
Hypertension, %						<0.001
No	4258 (68.4)	1310 (84.2)	1194 (76.7)	1008 (64.8)	746 (47.9)	
Yes	1966 (31.6)	246 (15.8)	362 (23.3)	548 (35.2)	810 (52.1)	
CKD, %						<0.001
No	5788 (93.0)	1451 (93.3)	1480 (95.1)	1442 (92.7)	1415 (90.9)	
Yes	436 (7.0)	105 (6.7)	76 (4.9)	114 (7.3)	141 (9.1)	
HUA, %						<0.001
No	5489 (88.2)	1480 (95.1)	1426 (91.6)	1334 (85.7)	1249 (80.3)	
Yes	735 (11.8)	76 (4.9)	130 (8.4)	222 (14.3)	307 (19.7)	
Fat intake, g/day	75.4 (54.1, 101.8)	75.2 (54.4, 101.7)	77.4 (56.4, 105.5)	72.8 (52.8, 98.0)	75.5 (53.8, 101.5)	0.003
Energy intake, kcal/day	2047.0 (1572.0, 2640.0)	2016.0 (1540.0, 2639.0)	2094.0 (1620.0, 2742.0)	2016.0 (1551.0, 2581.0)	2060.0 (1578.0, 2603.0)	0.006
Protein intake, g/day	80.2 (59.4, 104.8)	77.2 (57.5, 102.2)	83.4 (60.9, 109.9)	78.6 (58.9, 102.2)	80.6 (60.2, 104.1)	<0.001
Carbohydrate intake, g/day	244.4 (181.6, 319.9)	242.4 (180.3, 320.9)	251.7 (185.0, 329.3)	243.2 (182.8, 313.0)	242.3 (180.7, 316.3)	0.102
Fiber intake, g/day	15.9 (10.8, 22.6)	15.4 (10.8, 21.4)	16.1 (10.9, 23.4)	15.8 (10.7, 22.1)	16.3 (10.7, 23.6)	0.023

Note: The data are presented as a mean ± SD or median (IQR) for the skewed variables or as numbers (proportions) for the categorical variables. Abbreviations: VFA, visceral fat area; BMI, body mass index; WC, waist circumference; TG, triglycerides; HbA1c, hemoglobin A1c; TC, total cholesterol; HDL-C, high-density lipoprotein cholesterol; Cr, creatinine; eGFR, estimated glomerular filtration rate; ACR, albumin-to-creatinine ratio; CKD, chronic kidney disease; HUA, hyperuricemia; Q1, Q2, Q3, and Q4 are quartiles of VFA.

**Table 2:** Characteristics of the participants according to sex.

Characteristic	Total (*n* = 6224)	Male (*n* = 3345)	Female (*n* = 2879)	*p* Value
HUA, %				<0.001
No	5489 (88.2)	2817 (84.2)	2672 (92.8)	
Yes	735 (11.8)	528 (15.8)	207 (7.2)	
VFA, cm^2^	71.6 (48.2, 104.1)	79.8 (53.9, 112.6)	64.0 (39.7, 94.0)	<0.001
SUA, mg/dL	5.1 ± 1.3	5.8 ± 1.2	4.4 ± 1.0	<0.001

Note: The data are presented as a mean ± SD or median (IQR) for the skewed variables or as numbers (proportions) for the categorical variables.

**Table 3:** Multivariable-adjusted ORs and 95%CI of the VFA quantiles associated with HUA.

VFA, cm^2^	Unadjusted		Model 1		Model 2		Model 3	
	OR (95%CI)	*p* Value	OR (95%CI)	*p* Value	OR (95%CI)	*p* Value	OR (95%CI)	*p* Value
Per 10 cm^2^ increase	1.13 (1.11~1.15)	<0.001	1.19 (1.16~1.22)	<0.001	1.12 (1.09~1.16)	<0.001	1.10 (1.07~1.14)	<0.001
Q1 (≤48.24)	Ref		Ref		Ref		Ref	
Q2 (48.24–71.64)	1.78 (1.33~2.38)	<0.001	2.05 (1.52~2.75)	<0.001	1.61 (1.18~2.21)	0.003	1.51(1.10~2.08)	0.011
Q3 (71.64–104.06)	3.24 (2.47~4.25)	<0.001	4.36 (3.28~5.81)	<0.001	2.87 (2.03~4.04)	<0.001	2.50 (1.75~3.56)	<0.001
Q4 (>104.06)	4.79 (3.68~6.22)	<0.001	8.27 (6.09~11.23)	<0.001	4.57 (3.05~6.86)	<0.001	3.77 (2.47~5.75)	<0.001
*p* for trend	<0.001		<0.001		<0.001		<0.001	
Male								
Per 10 cm^2^ increase	1.09 (1.07~1.11)	<0.001	1.15 (1.12~1.18)	<0.001	1.07 (1.02~1.11)	0.002	1.06 (1.02~1.11)	0.006
Q1 (≤53.93)	Ref		Ref		Ref		Ref	
Q2 (53.93–79.82)	1.36 (1~1.86)	0.052	1.66 (1.2~2.29)	0.002	1.18 (0.83~1.68)	0.345	1.15 (0.80~1.65)	0.444
Q3 (79.82–112.64)	2.42 (1.82~3.23)	<0.001	3.59 (2.59~4.97)	<0.001	2.04 (1.37~3.04)	<0.001	1.88 (1.28~2.92)	0.003
Q4 (>112.64)	2.66 (2.00~3.54)	<0.001	4.94 (3.44~7.1)	<0.001	2.29 (1.42~3.71)	0.001	2.04 (1.24~3.38)	0.005
*p* for trend	<0.001		<0.001		<0.001		<0.001	
Female								
Per 10 cm^2^ increase	1.18 (1.14~1.22)	<0.001	1.21 (1.16~1.26)	<0.001	1.19 (1.12~1.26)	<0.001	1.19 (1.12~1.27)	<0.001
Q1 (≤39.70)	Ref		Ref		Ref		Ref	
Q2 (39.70–63.99)	2.24 (1.25~4.02)	0.007	2.33 (1.29~4.21)	0.005	2.20(1.18~4.1)	0.013	2.12 (1.13~3.97)	0.019
Q3 (63.99–94.02)	2.95 (1.68~5.19)	<0.001	3.18 (1.78~5.66)	<0.001	2.85 (1.46~5.57)	0.002	2.50 (1.26~4.95)	0.009
Q4 (>94.02)	7.06 (4.18~11.92)	<0.001	8.30(4.67~14.75)	<0.001	7.01 (3.28~14.98)	<0.001	5.51(2.52~12.03)	<0.001
*p* for trend	<0.001		<0.001		<0.001		<0.001	

Note: Model 1 is adjusted for age and race. Model 2 is adjusted for Model 1+ marital status, BMI, WC, smoking status, drinking status, vigorous recreational activity, and carbohydrate intake. Model 3 is adjusted for Model 1+ and Model 2+ TG, ACR, HbA1c, HDL-C, gout, protein intake, and hypertension. Ref, reference; VFA, visceral fat area; BMI, body mass index; WC, waist circumference; TG, triglycerides; ACR, albumin-to-creatinine ratio; HbA1c, hemoglobin A1c; HDL-C, high-density lipoprotein cholesterol.

**Table 4:** Threshold effect analysis of VFA and HUA in males using the two-piecewise linear regression model.

Threshold of VFA	OR (95%CI)	*p* Value	*p* for Log Likelihood Ratio Text
<104.79 cm^2^	1.015 (1.005~1.025)	<0.05	<0.001
≥104.79 cm^2^	1.002 (0.995~1.010)	0.52	

## Data Availability

Publicly available datasets were analyzed in this study. This data can be found here: http://www.cdc.gov/nchs/nhanes/.
